# Cardiac Rehabilitation After Open Heart Surgery: A Narrative Systematic Review

**DOI:** 10.3390/jcdd11110376

**Published:** 2024-11-20

**Authors:** Eleni Delimanoli, Olav Muurlink, Pavlos Myrianthefs, Anna Korompeli

**Affiliations:** 1 Department of Nursing, National and Kapodistrian University of Athens, 11527 Athens, Greece; pmiriant@nurs.uoa.gr (P.M.); akorompe@nurs.uoa.gr (A.K.); 2 School of Business and Law, Central Queensland University, Brisbane, QLD 4000, Australia; o.muurlink@cqu.edu.au; 3 ICU University Unit at “Agioi Anargyroi” General Hospital, School of Health Sciences, National and Kapodistrian University of Athens, 14564 Kifisia, Greece

**Keywords:** exercise therapy, rehabilitation, valve surgery, bypass graft, cardiac surgery, cardiac surgical procedures, heart surgery

## Abstract

Background: Postoperative cardiac rehabilitation (CR) programs are increasingly recommended by clinicians, but only a minority of patients who have undergone open heart surgery participate in such programs. Participation rates in postoperative CR, if anything, appear to be declining. This systematic review examines the effectiveness of post-operative CR and reveals possible participation barriers. Methods: A search of two scholarly databases for primary research papers published in the last decade examining the impact of post-operative CR was conducted and the resultant papers reviewed. Results: The 21 resulting studies revealed physiological functioning improvement and a reduction in mortality and readmission rates, while highlighting an enhancement in mental status. Some of the studies recognized the need for nutritional support and suggested that age, gender, access to CR centers, and socioeconomic variables impact participation in CR. Conclusions: Post-operative CR participation rates continue to decline despite increasing evidence of the value of the approach.

## 1. Introduction

Even though cardiovascular disease (CVD) claims 17.9 million lives a year [1], participation rates in formal cardiac rehabilitation (CR) programs following coronary artery bypass graft surgery (CABG) and valve surgeries remain low [2,3,4,5]. Despite evidence suggesting benefits of CR, uptake globally in the year 2000 was as low as 35% of eligible patients [6], with numbers even in developed country settings like the United Kingdom sitting at just 50% two decades later [7].

Among the benefits thought to ensue from CR are psychological (notably, anxiety and depression), and physiological outcomes (for example, a reduction in cardiac risk factors, such as high cholesterol levels, blood sugar levels, BMI, and systolic arterial pressure) [8]. There appears to be a negative relationship between the degree of engagement with CR sessions and morbidity risk [9]. As a result, CR programs are now recommended in most guidelines for post-operative cardiac patients [10]. The aim of this systematic review is to systematically present recent evidence on post-operative CR.

## 2. Materials and Methods

A search was performed using the PubMed and Scopus databases, the two largest databases from a health research perspective. We used the following keyword combinations: (“heart surgery” OR “thoracic surgery” OR “cardiac surgical procedure” OR “by-pass graft” OR “valve surgery”) AND (rehabilitation OR “physical rehabilitation” OR “psychological rehabilitation” OR “sexual rehabilitation” OR “exercise therapy”) NOT (children OR infant*) NOT (lung* OR esophagus OR pulmonary) NOT preoperative. A database search was performed from January 2023 to February 2023. Duplicate studies were removed from the review and the contents of the included studies were examined, and studies that were firewalled or not in English were excluded.

We included studies that were published in the full years 2013 to 2022. Files of candidate papers for inclusion were placed in a shared folder, with the first and fourth author screening papers independently for inclusion, conferring in cases in which agreement was not reached. Preoperative CR studies and studies involving patients who underwent combined heart and lung surgery were excluded. Only research that studied patients who had undergone cardiac surgery were included; those with mixed populations were excluded. The authors also excluded study protocols, conference papers, posters, letters to editors and editorials systematic reviews, meta-analyses, and observational studies, as well as any studies that examined non-human populations. Only primary research papers, including experimental, quasi, comparison, and cohort studies, were included in the final analysis.

**Figure 1:**
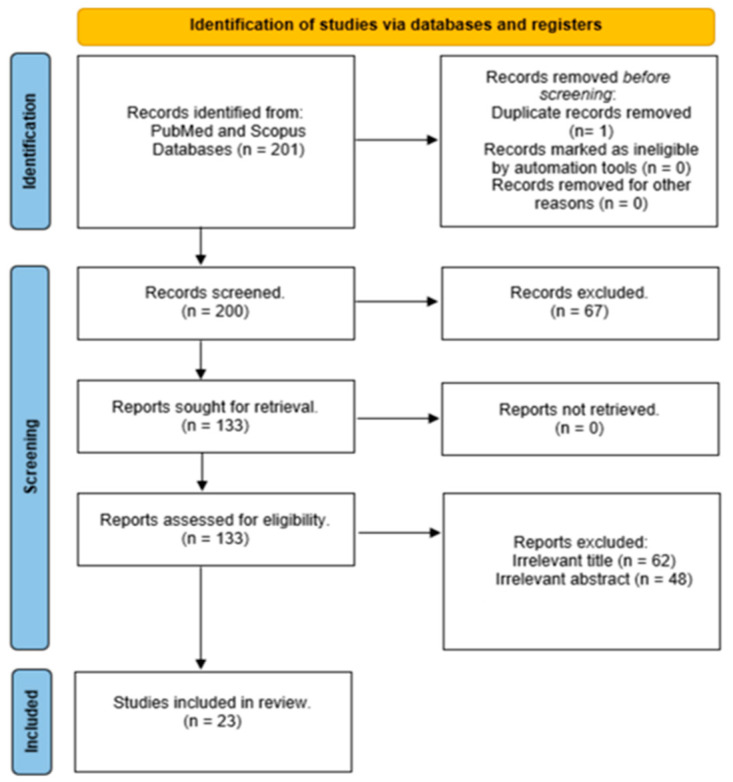
PRISMA diagram of literature search.

The present systematic review adhered to PRISMA guidelines, with the study selection process outlined in Figure 1. The initial search yielded 201 studies. After a review of the abstracts and full articles, the removal of duplicates, and a screening for appropriateness, 21 studies remained for review (see Figure 1).

The key characteristics of included studies are presented in Table 1. Methodologies included randomized controlled trials (7 studies), cross-sectional studies (5), cohort studies (4), and case–control studies (5). The review population included patients who underwent open-heart surgery, such as coronary artery bypass graft (CABG), heart valve surgery, or combined CABG and heart valve surgery. Most of the included studies focused on the second and third phases of CR (Phase 1: hospital inpatient, Phase 2: monitored outpatient, Phase 3: maintenance outpatient). A minority of studies analyzed the importance of nutritional support during the first rehabilitation phase. Research on the topic appears to be increasing, with most of the studies published in the last five years. Most exhibited a longitudinal design and were conducted at a single site.

Quality appraisal was performed according to the Critical Appraisal Skills Programme (CASP) guidelines. The CASP scale is a three-point scale, with studies either strongly, somewhat, or not exhibiting certain characteristics. Appendix A presents the results of the quality assessment.

## 3. Results

The majority of the papers in the review examined whether CR has measurable benefits. Most of the studies (including all seven of the RCTs) support the view that CR has statistically significant effects on health and well-being.

### 3.1. Mortality

Mortality was measured in seven studies, with five studies showing reduced mortality in CR patients [5,11,12,13,14], which was significant in two studies. Pollman et al.’s [14] retrospective study, even with a relatively small N (250), found a significant effect on mortality and long-term morbidity of CR after heart valve surgery. In the mean follow-up of 2.7 years, six deaths occurred. The cumulative hazard rate was higher, approximately tripled, among those not attending CR. However, that study did not assign participants randomly to CR or a control group, but instead relied on those who turned down, cancelled, failed to participate or dropped out of CR as the control. The cohort study by Goel et al. [12] initially included only 201 patients, and over a mean follow-up of 6.8 years, 86 deaths occurred. The study found that CR participation after combined heart valve and CABG surgery was associated with a significant reduction in long-term mortality. CR participation was also associated with a significant reduction in mortality, with an absolute risk reduction over 10 years of 14.5%. The mortality benefit of CR did not differ significantly in relation to age, sex, emergency status, or history of heart failure or arrhythmia. Again, the study’s weakness was the lack of adequate control, with patients not randomly allocated to the intervention. However, the authors conducted post hoc statistical analyses to strengthen their contention that CR did lead to a significant reduction in mortality.

The other three studies that examined mortality found significant effects. However, they lacked a non-CR control and did not explore the impact of CR itself. Yuen et al. [5] compared traditional and hybrid cardiac rehabilitation (CR) programs, for example, and found no evidence of a statistically significant difference in all-cause mortality between the approaches. Their findings suggest that the hybrid CR program, which involves a longer duration of rehabilitation and a home-exercise regimen, may be as effective as traditional CR programs in reducing mortality. In a case–control study by Ohkuma et al. [13], patients requiring nutritional support had an unadjusted operative mortality rate of 34% compared with the comparison group (2.7%). Additionally, the study notes that the in-hospital mortality rate associated with the need for nutritional support was greater than 30%. In another study that reported an effect on mortality, a single-blind, randomized, controlled trial [11], the main finding was that higher physical function, as assessed by the Short Physical Performance Battery (SPPB), was associated with a lower likelihood of mortality in patients undergoing heart valve surgery. A logistic regression analysis showed that a higher SPPB score predicted a reduced risk of mortality after six months of follow-up. This suggests that early inpatient (i.e., Phase 1) cardiac rehabilitation improves physical function and might contribute to positive recovery and potentially reduce the mortality rates in these patients.

Turning to the studies that failed to show an effect, Safdar et al.’s [4] retrospective study showed no statistically significant difference in major adverse cardiac outcomes, including mortality, at the 12-month post-operative mark between patients who participated in cardiac rehabilitation (CR) after coronary artery bypass grafting (CABG) surgery and those who did not participate in CR. In the randomized CopenHeartVR clinical heart valve surgery trial [15], at 24-month follow-up, 3% of the patients in both the intervention and the control groups died. The combined cumulative incidences of overall readmission and mortality showed that 68% of the patients in the intervention group and 75% in the control group were readmitted or died after 24 months. Again, there was no statistically significant difference in the overall readmission or mortality between the two groups, despite the data showing reductions in readmission rates and mortality rates at up to 12 months post-intervention in the intervention group.

### 3.2. Readmissions and Clinical Parameters

A minority of studies [15,18] examined patients over a sufficient period of time to be able to conclude whether or not CR had enduring positive effects. In each case, changes observed immediately post-CR or while CR was underway were tracked for time periods up to 24 months post-surgery CR. Readmission trended towards reductions at the 3-, 6-, and 12-month marks, post-surgery [15]. In another study, deteriorating clinical parameters were less likely amongst the CR participants at the 12-month follow-up mark [18].

### 3.3. Physical Functioning

Relative to the ambiguity of the mortality studies, examinations of the impact of CR on functional capacity tended to be more consistent [15,19,20]. In a cross-sectional study, Savage et al. [21] found that peak VO2 increased by a mean of 19.5% from baseline by the time cardiac rehabilitation programs were completed. Within the group of patients who underwent valve surgery, the peak VO2 increased by a mean of 22.0%. The percentage increase in peak VO2 was similar among the three types of valvular abnormalities (mitral, aortic, and mitral + aortic). More specifically, the key findings for physical functioning and handgrip strength were lower in both groups of valve patients than in individuals who underwent coronary artery bypass grafting (CABG). By contrast, the Medical Outcomes Study, 36-Item Short Form Survey (MOS-SF-36) scores, which indicate self-reported physical functioning, were similar among all surgical groups of patients. In the randomized CopenHeartVR clinical trial [15], conducted over a 12-month period, both the intervention and the control group showed an increase in physical capacity measured by peak oxygen uptake (VO2 peak) after heart valve surgery. Both groups showed improvement in physical functioning, as measured by the 6-minute walk test and sit-to-stand test from baseline to 12 months, with no statistically significant differences in the effect over time between the groups. The data did show reductions in readmission rates at up to 12 months post-intervention in the intervention group; however, there was no statistical significance.

Finally, in one of the more robust studies, a single-blind, randomized, controlled trial study by Xue et al. [20], early (Phase 1) cardiac rehabilitation in patients with heart valve surgery was found to be effective in improving physical function, as assessed by the SPPB and the 6-minute walking test. Furthermore, this study found that an improvement in VO2 peak, which predicts readmission for cardiovascular disease and a reduction in mortality, was found in patients undergoing cardiac rehabilitation after heart valve surgery. Another finding was that higher physical function (measured by the SPPB), associated with participation in cardiac rehabilitation, is associated with lower mortality rates in patients with heart valve surgery.

### 3.4. Psychological Effects

Six papers measured psychological impacts [15,16,17,18,19,20,21,22] using a range of measures. A US study that tracked depression through a CR program showed improvements in both coronary artery bypass graft (CABG) and heart valve (HV) surgical patients [21]. Two research teams using the same Hospital Anxiety and Depression Scale found no change following a study of cardiac rehabilitation [20], a result confirmed in the CopenHeartVR data study by Sibilitz’s team [15]. The latter study found that almost a quarter of patients had anxiety at baseline, in line with studies showing that physical disease burden commonly has psychological correlates. In an RCT comparing three months of extended CR with more typical shorter (one-month) care using the Depression Anxiety and Stress Scale, it was found that depression, anxiety, and stress all dropped significantly in both the short and the long version of CR [21]. Two studies primarily focused on the psychological impact of CR. Pourafkari et al.’s [22] small longitudinal study found statistically significant reductions in both anxiety and depression in patients with CR over the course of an eight-week program. Modica et al.’s [20] study of 1179 CABG and 737 VR patients revealed that CABG patients tend to exhibit higher levels of anxiety about their health progress. Both subgroups showed high anxiety and depression levels, suggesting the need for psychological support as a component of CR programs.

### 3.5. Nutritional Effects

The interaction between nutrition and CR was a focal point of interest in three studies [13,23,24]; moreover, nutritional status was recorded in a number of additional studies due to the recognition of the role of nutrition in determining health status in postoperative health. Tramarin et al.’s retrospective Italian study examined iron deficiency in patients introduced to CR [24], found that iron deficiency was associated with reduced exercise and quality of life measures. Boban et al. [23] and Ohkuma et al. [13] examined the need for nutritional support among in-patient CR participants, as well as validating the Johns Hopkins Hospital Nutrition Support Scale in identifying at-risk patients. A previous study showed that nutritional risks were commonly present in patients presenting for rehabilitation post-cardiac surgery, recommending nutritional risk screening, a finding that also emerged in Tramarin et al.’s study [24]. Malnutrition appears to be related to increased length of hospital stay and readmission rates, as well as mortality. Paradoxically, overweight at-risk patients have more opportunities for improvement from CR, with advanced patient age being positively associated with body weight loss [23,25]. These studies also showed that patients with diabetes were less likely to lose weight [23,25], while patients with high blood urea and creatinine levels were more likely to do so [23].

### 3.6. Barriers and Participation Rates in CR

A major barrier to participation is the lack of medical attention to reinforce participation in CR, and, more generally, effective communication with patients [26]. CR participation rates vary sharply by country, which impacts on the degree to which results can be generalized. Among the studies captured in this review, participation rates ranged from almost 80%, in the Netherlands [14], to much lower levels in parts of the USA: 55% was recorded in Connecticut, with the rate falling as low as 47% in Minnesota [4,12]. Reported rates are even lower in Australia [2], suggesting that participation is not a function of level of healthcare infrastructure development. Pollman et al.’s [14] high-end 80% participation rate may have been overestimated due to increased participation demand. Certainly, as the authors of that study note, “a few patients with residency outside the catchment area asked for a referral”. However, those living at greater distances from the facilities and older patients were less likely to accept referral. More broadly, women appear to be less likely to participate in CR [9,20,22]; as well as advanced-age patients [14,26]. Lower CR participation rates have been reported in racial or ethnic minority group patients [4]. Socioeconomic factors also played a part: patients living alone or those with lower income were less likely to participate in CR sessions [26]. These socioeconomic factors are likely to interact with factors such as the geographical accessibility of CR centers: geographical distance is a common barrier for patient participation [4,5]. Social isolation is also likely to be associated with a lack of family and psychological support, which are possible participation barriers [4,5].

### 3.7. Various Types of CR

A range of studies have examined the differences between CR implementations: early versus late enrolment [30]; home-based versus hospital or clinic-based CR [11]; resistance-based versus aerobic-based CR [27]; extended CR versus traditional-length treatment [21]; yoga-based versus traditional CR [28]; and hybrid versus conventional center-based approaches [5]. The burden of evidence suggests that not only does CR have a durable, significant and positive impact on a broad range of patient outcomes, but that particular types of CR have superior outcomes (with some evidence collectively showing that yoga-based, resistance-based, and hospital-based interventions are superior) [15,27,28]. Evidence also suggests that some efficiencies can be achieved in delivering CR. For example, no significant difference was found in a relatively small study comparing early and late enrolment in CR [30], and relatively few resource-intensive hybrid programs might be just as effective as typical CR interventions [5].

## 4. Discussion

This systematic review supports the effectiveness of CR in terms of physical and psychological measures. It identifies a number of participation barriers, notably those related to gender, age, and socioeconomic factors, and, interestingly, points to a previously unacknowledged function of CR, in offering prognostic assistance to clinicians. The evidence on mortality differences is not particularly strong, with a range of studies with different approaches producing either relatively small but statistically significant effects, or non-significant results. These findings nevertheless collectively suggest that exercise-based cardiac rehabilitation after heart valve surgery can provide short-term benefits in terms of physical capacity, but long-term effects on physical functioning remain unclear.

There was a gender bias in the study samples, with most of the CR studies having more male participants, with males being more likely to present with cardiac pathology [4]. Female patients were less likely to participate in CR programs per se, in part explaining the gender gap in these studies. Safdar et al. [4], from the US, revealed in their study that “annual mortality and rates of adverse events were low and similar in men and women” (page 853). However, this is not a universal finding. Bianchi et al. [31], for example, studying an Italian sample with a similar prevalence of males, found a benefit of CR amongst women, but showed a differentially lower benefit of CR. The authors suggest that women present with atypical (for the whole population of those available for CR) symptoms and circumstances including, for example, smaller coronary arteries and lower plaque levels. However, social roles may also play a part in determining diagnosis and treatment. While females have lower rates of cardiovascular disease, they experience higher mortality rates and significantly poorer prognoses after acute cardiovascular events.

Patients who had combined CABG and heart valve surgery appeared to show, not surprisingly, elevated post-operative mortality relative to patients who underwent CABG surgery alone [12]. Researchers found that patients who underwent combined CABG/heart valve surgery nevertheless benefitted from CR, showing lower mortality rates. CR was also beneficial in patients who underwent CABG surgery and mitral valve replacement [12]. Studies remain unclear on the mortality and readmission rates of patients who undergo heart valve surgery and participate in CR programs. Only two studies explored this group, with one showing mortality and readmission rates declining, but only within the 12-month CR [15], while the other [32] showed a statistically significant effect.

Increased functioning has been observed in most post-operative cardiac patients after CR program completion [4,20,30,33]. Improved functionality in itself leads to higher survival rates [15,27]. The research suggests that more work is required to assess the efficacy of different forms of CR. Extended length of outpatient CR sessions did not appear to be beneficial, for example, in one study [21]. Elsewhere, researchers found that while prolonged CR may be commonly used to improve quality of life [26], shorter programs also demonstrate VO2 max increases after CR sessions [12,20,21], with VO2 max increases associated with a significant decrease in mortality rates [20]. One study found that inpatient CR sessions proved more effective compared to home-based sessions [18]. However, it is important to note that, increasingly, programs are hybrid, using combinations of clinic-based and home-based interventions, making the real-world study of such differences quite challenging.

CR can reduce frailty and is considered a safe exercise approach, even for older patients, particularly in the first four months post-operation [18]. CR sessions included aerobic exercises and, rarely resistance training [4,11,14,21,27]. Shorter program options include exclusively aerobic and calm exercises, such as walking, static cycling, and breath relaxation [22,28,30]. Resistance exercises are commonly introduced only after the sixth post-operative week [19,27]. The benefits of CR as exercise are well supported by the literature. Muscle strength and body balance were remarkably improved by CR [18,27]. Yoga exercise was found to reduce the pre-existing CVD risk factors (ejection fraction, BMI, glucose, and blood lipids) [28], while CR exercise more generally appears to reduce atherosclerosis and thrombosis development [18]. Resistance training appears to reduce inflammation and slows cognitive decline. Researchers, in fact, observed a broader improvement in cognitive function, especially in patients who trained more with resistance exercises [27].

Turning to psychological factors, it is likely that psychological disorders have a two-way causal relationship with cardiovascular disease. Stress and anxiety contribute to CVD [33] and depression is frequently observed in patients admitted for cardiac surgery [22]. If CR becomes a dominant remedial approach, it is likely that this will have broader positive psychological outcomes. CR appears to offer a clear pathway to reduce stress and depression levels, especially after CABG [22]. According to recent data, exercise offers broader well-being improvements [21] and reduces anxiety sensitivity [3]. Depression is a major mortality factor that leads to decreased functional capacity and increased readmission rates in patients undergoing CABG [28]. Depression and stress are likely to appear post-operatively after CABG and heart valve surgery [21,29]. CR thus offers a promising psychological and physical treatment.

Turning to nutrition, there are, again, plausible two-way effects. Open heart surgery in itself provokes elevated interleukin 6, which in turn causes transferrin deficiency, leading to absolute or functional iron deficiency [24]. Iron deficiency and poor nutrition affect patients’ quality of life [13,24]. Poor nourishment causes an increase in mortality rates, readmission rates, and length of hospital stays [13,23]. In addition, it causes morbidity and is associated with poorer psychological states [23]. Weight reduction amongst overweight patients is partly predicted by initial body mass [23,25]. Therefore, monitoring nutrition and providing nutritional support through patient education during CR sessions is recommended. As Bianchi et al. note, BMI represents a key point of intervention for CR programs, unlike gender and age [31], although there is some evidence that weight loss is not always an advantage in patients with heart disease [34].

Nurses clearly have a key role to play in how CR is communicated to patients, as well as how programs are enacted and monitored. Nurses are often key stakeholders in all phases of cardiac recovery and inpatient and outpatient cardiac rehabilitation programs. Nurses support patient participation, with nurse advocacy found to highly impact patient enrolment [26]. CR nurses have a strategic role in overcoming barriers to ongoing participation, perhaps most critically when it comes to clear communication, which supports participation rates. They also have a role in addressing access gaps, as well as dealing with medical prescription issues, and tailoring nutritional advice and support. A lack of specialized CR nurses in CR teams, noted in two studies [2,26], is thus concerning. CR teams should integrate nurses for comprehensive care and communication. Finally, further studies exploring the role of nurse-led rehabilitation programs are required, to better optimize the integration of nurses in CR response.

### Limitations

This systematic review has a number of limitations. The review was limited to studies published in English in the last 10 years, and was limited to two scholarly databases, although these databases did have a broad reach in health research. In addition, patient comorbidities remain an open question for researchers. Comorbidities may negatively affect patient participation and exercise output. Finally, the heterogeneity of the included studies, employing a range of measures, created difficulty in the reconciliation of the results. Collectively, these studies offer promise, but there is a distinct need for further RCT studies to refine our understanding of effective CR interventions. There is sufficient evidence to suggest that CR is a worthwhile post-operative intervention. However, further work evidencing the tailoring of CR for particular sub-populations, such as by gender, age, and socioeconomic brackets, is clearly required. Furthermore, improving our understanding of the role of nutrition and measures to increase access, participation, and retention in CR programs is strongly indicated, in keeping with the advice of the American Association of Cardiovascular and Pulmonary Rehabilitation.

## 5. Conclusions

The findings of this systematic review suggest the value of CR for post-operative patients across a number of key variables. While the evidence is not overwhelming, stronger studies suggest that CR contributes to functional improvement at the physical and psychological levels, and it is plausible that larger, better-controlled studies would show a more consistent effect on mortality. Functional improvement occurs through rehabilitation exercises, education, and consultations. Rehabilitation exercise increases VO2 max and endurance and decreases mortality and readmission rates. CR also appears to have some efficacy in decreasing anxiety and depression levels post-operatively. A number of correlational studies provide some evidence that nutritional status is worth monitoring in open heart surgery patients, and nutritional support in combination with CR may have promise as a therapeutic approach. In general, while this review covers studies that do not directly explore the role of nurses, it offers evidence that nurses play a critical role in the CR team, with evidence collectively suggesting the importance of reliable program control, communication, and tailored, monitored programs. This suggests that nurses are key stakeholders in the enrolment and retention of patients in rehabilitation programs.

## Figures and Tables

**Table 1:** Main characteristics of the reviewed studies.

Reference	Country	Type of Study	Focus	Sample Description	Outcomes	Results (Statistically Significant, Unless Otherwise Stated)	Limitations
Safdar et al. (2022) [4]	U.S.A.	Cohort study	Examined clinical data and complications during CR to 12 months post-commencement	420 CABG patients	Mortality and postoperative complications	Readmission rate reduction, strength and fitness increased in absolute terms, but no statistical significance in mortality rates	Small cohort study
Yuen et al. (2022) [5]	Canada	Case-control study	Compared hybrid CR approach with typical CR care	477 patients (326 patients in typical CR care and 151 patients in hybrid CR)	Participation barriers, major adverse effects, exercise tolerance, functionality	Hybrid program is less resource intense, and no significant differences emerged between hybrid and typical CR	Small sample size means low power for study on certain outcomes (such as mortality), no blinding, all participants showing a good fitness status, design does not include consideration of confounds
Butkuviene et al. (2022) [11]	Lithuania	RCT	Compared supervised home and hospital exercise with non-supervised exercise	100 patients (1:1 randomization)	Functional capacity before and after CR	Pace rhythm and kinetic energy improved. Home-based exercise training did not result in significant change	One-third of the data were lost due to elder patients
Goel et al. (2015) [12]	U.S.A.	Cohort study	Examined the influence of CR on mortality rates	201 patients experiencing combined heart valve and coronary artery bypass grafts	Mortality rates, risk of mortality, patient participation	Patients not randomly assigned, but increased survival rates amongst those who did CR. CR participation was more beneficial for mitral valve procedure patients	Concluded only one region of the U.S.A., small sample
Ohkuma et al. (2017) [13]	U.S.A.	Case-control study	Examined the need of postoperative nutritional support	1056 patients	Biochemical measures, operation duration, mortality rates, hospital stay, discharge rates	Increased mortality rates and hospital stay for patients who need nutritional support	Confounds not considered, single site study, no preoperative data
Pollman et al. (2017) [14]	Denmark	Case-control study	Evaluated the VO_2_ max consumption and the distance reached at 6MWT	146 heart valve surgery patients	Exercise tolerance, mortality, and readmission rates, clinical complications	VO_2_ max and distance reached showed significant improvement, increased clinical complications at patients that did not participate in CR; age > 75 was predictive of non-participation	Lack of data about the social and financial patient status, lack of data from preoperative health status, did not encounter stress and depression
Sibilitz et al. (2022) [15]	Denmark	RCT	Compared 12 weeks CR that included consulting and educational sessions with standard care	147 heart valve surgery patients (1:1 randomization)	Functional capacity, physical and psychological health, quality of life, readmission, and mortality rate	Mortality and readmission rate decline observed at 12 months post CR, functional capacity improvement only after 4 months of CR, no changes in physical and psychological health	No blinding, no primary measures of variables
Aronov et al. (2019) [16]	Russia	RCT	Compared CR that included individualized exercise, education, and home exercise with typical CR care	36 CABG, male patients (1:1 randomization)	Exercise tolerance, risk of CVD factors, clinical parameters	Reduced risk of CVD factors, clinical parameters, and physical functionality improvement. Improved physical endurance maintained at 12 months post-intervention commencement	Small sample size, one-center study, only male participants
Pack et al. (2015) [17]	U.S.A.	Case-control study	Compared early CR with traditional postoperative care	112 CABG, 69 heart valve and 59 myocardial infraction patients	Length of hospital stay, major adverse effects, readmission rates and patient participation (early or late in CR program	No significant difference between early and traditional (late) enrolment in CR	Small sample size, not possible randomization, retrospective study
Xue et al. (2022) [18]	China	Randomized Control Study (RCT)	Compared early and traditional CR	87 patients (44 intervention group, 43 control group) after heart valve operation	Functional capacity, psychological functionality, mortality rates	Enduring and improved and functional capacity, decreased mortality rates	Included only heart valve surgery patients, Data from questionnaires- possible overestimation
Savage et al. (2015) [19]	U.S.A.	Cross-sectional study	Examined exercise performance in CABG, heart valve and combined surgery patients	313 patients	VO_2_ max, age, gender, body weight, BMI, number of CR sessions, comorbidities, depression	Aerobic capacity, muscle strength, functionality, and depression improvement	One-center study, Observational study without randomization, relative exercise intensity not reported
Modica et al. (2018) [20]	Italy	Cross-sectional study	Compared anxiety and depression levels of CABG patients with the levels of heart valve patients	1179 CABG patients, 737 heart valve patients	Anxiety and depression levels, physical and psychological concern	CABG patients had increased physical and psychological concern, similar anxiety and depression levels in both patient groups	CR not the focus of the study; ‘ross-sectional’. No preoperative data available, data collection only in the early postoperative period, short-term design does not permit examining enduring effects
Pakrad et al. (2022) [21]	Iran	RCT	Compared 3 months extended CR with typical CR care	88 CABG patients (1:1 randomization)	Quality of life, functionality, readmission rates, stress and depression levels	Functionality, quality of life and wellbeing improvement, readmission rates decline. Results sustained 3 months post-CR	Single center study, no blinding possible
Pourafkari et al. (2016) [22]	Iran	Cross-sectional study	Examined the effect of CR on anxiety and depression levels	40 CABG patients	Anxiety and depression levels before and after CR	Decreased anxiety and depression levels after CR sessions, increased self-care	Cross-sectional study
Boban et al. (2013) [23]	Croatia	Cohort study	Researched the increase in nutritional risks through in-patient rehabilitation	145 patients	Body weight loss, nutritional risk	High nutritional risk for all postoperative cardiac patients, related with renal failure and advanced age	Patients that were contraindicated for being included, were excluded. Study not focused on CR
Tramarin et al. (2017) [24]	Italy	Cohort study	Examined the factors that contribute to absolute-functional iron deficiency in CR	339 patients	Iron deficiency factors, deficiency complications	Iron deficiency negatively associated with exercise, safety and quality of life, recommends need for certain nutrition, exercise and medicine in CR	Retrospective study, possible data leak, data for preoperative iron status unavailable
Wilkinson et al. (2021) [25]	United Kingdom	Case-control study	Examined patient characteristics that contributed to easier body weight loss	29,601 patients	Gender, age, fitness level, pain, social status, participation	Increased body weight loss in patients with advanced age, employed workers, smokers who did not cease smoking and male patients. Pain, lower initial fitness, lower SES geographical locations predicted less weight loss	Short study timeframes, unclear whether diet instructions and the patient support given in CR
Conijn et al. (2022) [26]	The Netherlands	Cross-sectional study	Studied enrollment and referral rates in CR	364 patients	Enrollment rates at the second phase of CR, causes of non-enrollment and participation	Older female patients less likely to be referred and enroll. Those who live alone and have lower income were also less likely to enroll	Single center study, 50% CR completion rate, no control
Pengelly et al. (2022) [27]	Australia	RCT	Compared 12 weeks early CR that included resistance exercises with aerobic CR	39 patients (1:1 randomization)	Postoperative cognitive function at 14 weeks and at 6 months, strength, and balance	Better cognitive function (Alzheimer’s Disease Assessment Scale) for patients that did resistance exercises for 14 weeks, 53% of the aerobic-based rehabilitation control group experienced cognitive decline compared to 0% in the resistance training group	Small sample size, possible type-2 error, pilot study
Raghuram et al. (2014) [28]	India	RCT	Compared yoga CR with typical CR care	250 CABG, male patients	Ejection fraction (EF), stress- depression levels, BMI, blood sugar and blood lipids	Yoga approach showed widespread advantages, including in EF improvements in those with abnormal EF baseline, and overall improvements in BMI, perceived stress, anxiety, depression and negative affect. HDL, LDL and VLDL improvements in yoga group for those with abnormal baseline levels	Increased sample search duration, patients with very low EF were excluded, 40% of the patients exited study within 1 year
Racca et al. (2015) [29]	Italy	Cross-sectional study	Evaluated CR patients with International classification of Functioning Disability and Health (ICF) scale	50 CABG and heart valve surgery patients	Functional capacity, body structure, patient participation, environmental factors	First study to use ICF scale, providing support for the scale in major heart surgery setting	Did not examine functionality in heart transplant surgery, patients, no comparison group

## Data Availability

No new data were created.

## Appendix A

The results are summarized in Table A1, Table A2 and Table A3. Note that none of the RCT studies, only two cohort studies, and two case–control studies were judged to be of high quality.

**Table A1:** Summarized quality appraisal of cohort studies based on CASP checklist.

Study	Q1	Q2	Q3	Q4	Q5 (a)	Q5 (b)	Q6 (a)	Q6 (b)	Q7	Q8	Q9	Q10	Q11	Q12	Quality
Safdar et al. [4]	Yes	Yes	Yes	Cannot tell	Cannot tell	No	Yes	Yes	Yes	Yes	Yes	Yes	Yes	Yes	Medium
Goel et al. [12]	Yes	Yes	Yes	Yes	Yes	Yes	Yes	Yes	Yes	Yes	Yes	Cannot tell	Yes	Yes	High
Savage et al. [19]	Yes	Yes	Yes	Yes	Cannot tell	Yes	Cannot tell	Cannot tell	Yes	Yes	Yes	Yes	Cannot tell	Yes	Medium
Modica et al. [20]	Yes	Yes	Yes	Cannot tell	Yes	Yes	Cannot tell	No	Yes	Cannot tell	Yes	Yes	Yes	Yes	Medium
Pourafkari et al. [22]	Yes	Cannot tell	Cannot tell	Yes	Yes	No	Cannot tell	No	Yes	Yes	Cannot tell	Yes	Cannot tell	Yes	Low
Boban et al. [23]	Yes	Yes	Yes	Yes	Yes	Yes	Cannot tell	Cannot tell	Yes	Yes	Yes	Yes	Yes	Yes	High
Tramarin et al. [24]	Yes	Yes	Yes	Can’t tell	No	Can’t tell	Can’t tell	No	Can’t tell	Yes	Can’t tell	Yes	Can’t tell	Yes	Low
Conijn et al. [26]	Yes	Yes	Yes	Yes	Cannot tell	No	Yes	Yes	Yes	Yes	Yes	Yes	Yes	Yes	Medium
Racca et al. [29]	Yes	Yes	Yes	Cannot tell	Cannot tell	Yes	Yes		Yes	Cannot tell	Yes	Yes	Cannot tell	Yes	Low

Quality assessment questions: Q1 = “Did the study address a clearly focused issue?”; Q2 = “Was the cohort recruited in an acceptable way?”; Q3 = “Was the exposure accurately measured to minimize bias”; Q4 = “Was the outcome accurately measured to minimize bias?”; Q5 (a) = “Have the authors identified all important confounding factors ?”; Q5 (b) = “Have they taken account of the confounding factors in the design and/or analysis?”; Q6 (a) = “Was the follow-up of subjects complete enough?”; Q6 (b) = “Was the follow-up of subjects long enough?”; Q7 = “What are the results of this study?”; Q8 = “How precise are the results?”; Q9 = “Do you believe the results?”; Q10 = “Can the results be applied to the local population?”; Q11 = “Do the results of this study fit with other available evidence?”; Q12 = “What are the implications of this study for practice?”. Answer key: “Yes” or strong, “No” or weak, “Cannot tell” or uninterpretable.

**Table A2:** Summarized quality appraisal of case control studies based on CASP checklist.

Study	Q1	Q2	Q3	Q4	Q5	Q6 (a)	Q6 (b)	Q7	Q8	Q9	Q10	Q11	Quality
Yuen et al. [5]	Cannot tell	Yes	Yes	Cannot tell	Cannot tell	Yes	No	No	Yes	No	Cannot tell	Cannot tell	Low
Ohkuma et al. [13].	Yes	Yes	Yes	Yes	Yes	Yes	No	Cannot tell	Yes	Yes	No	No	Low
Pollman et al. [14].	Cannot tell	Yes	Yes	Yes	Cannot tell	Yes	Cannot tell	Yes	Yes	Yes	Yes	Yes	High
Pack et al. [17]	Yes	Yes	Yes	Cannot tell	Yes	Yes	No	Yes	Yes	Yes	Yes	Yes	Medium
Wilkinson et al. [25]	Yes	Yes	Yes	Yes	Yes	Yes	Cannot tell	Yes	Yes	Yes	Yes	Yes	High

Quality assessment questions: Q1 = “Did the study address a clearly focused issue?”; Q2 = “Did the authors use an appropriate method to answer their question?”; Q3 = “Were the cases recruited in an acceptable way?”; Q4 = “Were the controls selected in an acceptable way?”; “Q5 = “Was the exposure accurately measured to minimize bias?”; Q6 (a) = “Aside from the experimental intervention, were the groups treated equally?”; Q6 (b) = “Have the authors taken account of the potential confounding factors in the design and/or in their analysis?”; Q7 = “How large was the treatment effect?”; Q8 = “How precise was the estimate of the treatment effect?”; Q9 = “Do you believe the results?”; Q10 = “Can the results be applied to the local population?”; Q11 = “Do the results of this study fit with other available evidence?”. Answer key: “Yes” or strong, “No” or weak, “Cannot tell” or uninterpretable.

**Table A3:** Summarized quality appraisal of RCT’s based on CASP checklist.

Study	Q1	Q2	Q3	Q4 (a)	Q4 (b)	Q4 (c)	Q5	Q6	Q7	Q8	Q9	Q10	Q11	Quality
Butkuviene et al. [11]	Yes	Yes	Yes	Yes	Cannot tell	Yes	Yes	No	Cannot tell	Yes	Yes	Yes	Yes	Medium
Sibilitz et al. [15]	Yes	Yes	No	Yes	No	Yes	Yes	No	Yes	Yes	Cannot tell	Cannot tell	Yes	Low
Aronov et al. [16]	Yes	Cannot tell	Yes	Cannot tell	Cannot tell	Cannot tell	No	No	Yes	Yes	Yes	Yes	Yes	Low
Xue et al. [18]	Yes	Yes	Cannot tell	Yes	No	Yes	Yes	No	Yes	Yes	Cannot tell	Yes	Yes	Medium
Pakrad et al. [21]	Yes	Yes	Yes	No	No	Yes	Yes	No	Cannot tell	Yes	Yes	Yes	Yes	Low
Pengelly et al. [27]	Yes	Yes	No	No	No	No	Yes	No	Yes	Yes	Yes	Yes	Yes	Low
Raghuram et al. [28]	Yes	Yes	No	No	No	Yes	Yes	No	Yes	Yes	Yes	Yes	Yes	Low

Quality assessment questions: Q1 = “Did the study address a clearly focused research question?”; Q2 = “Was the assignment of participants to interventions randomized?”; Q3 = “Were all participants who entered the study accounted for at its conclusion?”; Q4 (a) = “Were the participants ‘blind’ to intervention they were given?”; Q4 (b) = “Were the investigators ‘blind’ to the intervention they were giving to participants?”; Q4 (c) = “Were the people assessing/analyzing outcome/s ‘blinded’?”; “Q5 = “Were the study groups similar at the start of the randomized controlled trial?”; Q6 = “Apart from the experimental intervention, did each study group receive the same level of care (that is, were they treated equally)?”; Q7 = “Were the effects of intervention reported comprehensively?”; Q8 = “Was the precision of the estimate of the intervention or treatment effect reported?”; Q9 = “Do the benefits of the experimental intervention outweigh the harms and costs?”; Q10 = “Can the results be applied to your local population/in your context?”; Q11 = “Would the experimental intervention provide greater value to the people in your care than any of the existing interventions?”. Answer key: “Yes” or strong, “No” or weak, “Cannot tell” or uninterpretable.

## References

[1] World Health Organization (WHO). https://www.who.int/.

[2] Nasrawi D., Latimer S., Massey D., Gillespie B.M. (2023). Delivery, barriers, and enablers to patient participation in inpatient cardiac rehabilitation following cardiac surgery: An integrative review. Aust. Crit. Care.

[3] Osuji E., Prior P.L., Suskin N., Frisbee J.C., Frisbee S.J. (2022). The relationship between anxiety sensitivity and clinical outcomes in cardiac rehabilitation: A scoping review. Am. J. Prev. Cardiol..

[4] Safdar B., Mori M., Nowroozpoor A., Geirsson A., D’Onofrio G., Mangi A.A. (2022). Clinical profile and sex-specific recovery with cardiac rehabilitation after coronary artery bypass grafting surgery. Clin. Ther..

[5] Yuen T., Buijs D.M., Hong Y., Van Damme A., Meyer T.C., Nagendran J., Gyenes G.T. (2022). Comparing the effectiveness of two cardiac rehabilitation exercise therapy programs. CJC Open.

[6] Bethell H. (2000). The BACR database of cardiac rehabilitation units in the UK. Coron. Health Care.

[7] Harrison A.S., Gaskins N.J., Connell L.A., Doherty P. (2020). Factors influencing the uptake of cardiac rehabilitation by cardiac patients with a comorbidity of stroke. IJC Heart Vasc..

[8] Dalal H.M., Doherty P., Taylor R.S. (2015). Cardiac rehabilitation. BMJ.

[9] Medina-Inojosa J.R., Grace S.L., Supervia M., Stokin G., Bonikowske A.R., Thomas R., Lopez-Jimenez F. (2021). Dose of cardiac rehabilitation to reduce mortality and morbidity: A population-based study. J. Am. Heart Assoc..

[10] Ubeda-Tikkanen A., Gauthier N.S. (2019). Cardiac rehabilitation and exercise training. Exercise Physiology for the Pediatric and Congenital Cardiologist.

[11] Butkuviene M., Tamuleviciute-Prasciene E., Beigiene A., Barasaite V., Sokas D., Kubilius R., Petrenas A. (2022). Wearable-based assessment of frailty trajectories during cardiac rehabilitation after open-heart surgery. IEEE J. Biomed. Health Inform..

[12] Goel K., Pack Q.R., Lahr B., Greason K.L., Lopez-Jimenez F., Squires R.W., Zhang Z., Thomas R.J. (2015). Cardiac rehabilitation is associated with reduced long-term mortality in patients undergoing combined heart valve and CABG surgery. Eur. J. Prev. Cardiol..

[13] Ohkuma R.E., Crawford T.C., Brown P.M., Grimm J.C., Magruder J.T., Kilic A., Suarez-Pierre A., Snyder S., Wood J.D., Schneider E. (2017). A novel risk score to predict the need for nutrition support after cardiac surgery. Ann. Thorac. Surg..

[14] Pollmann A.G.E., Frederiksen M., Prescott E. (2017). Cardiac rehabilitation after heart valve surgery: Improvement in exercise capacity and morbidity. J. Cardiopulm. Rehabil. Prev..

[15] Sibilitz K.L., Tang L.H., Berg S.K., Thygesen L.C., Risom S.S., Rasmussen T.B., Schmid J.P., Borregaard B., Hassager C., Køber L. (2022). Long-term effects of cardiac rehabilitation after heart valve surgery—Results from the randomised CopenHeartVR trial. Scand. Cardiovasc. J..

[16] Aronov D., Bubnova M., Iosseliani D., Orekhov A. (2019). Clinical efficacy of a medical centre- and home-based cardiac rehabilitation program for patients with coronary heart disease after coronary bypass graft surgery. Arch. Med. Res..

[17] Pack Q.R., Squires R.W., Lopez-Jimenez F., Lichtman S.W., Rodriguez-Escudero J.P., Lindenauer P.K., Thomas R.J. (2015). Participation rates, process monitoring, and quality improvement among cardiac rehabilitation programs in the United States: A national survey. J. Cardiopulm. Rehabil. Prev..

[18] Xue W., Xinlan Z., Xiaoyan Z. (2021). Effectiveness of early cardiac rehabilitation in patients with heart valve surgery: A randomized, controlled trial. J. Int. Med. Res..

[19] Savage P.D., Rengo J.L., Menzies K.E., Ades P.A. (2015). Cardiac rehabilitation after heart valve surgery: Comparison with coronary artery bypass graft patients. J. Cardiopulm. Rehabil. Prev..

[20] Modica M., Castiglioni P., Minotti A., Faini A., Racca V., Ferratini M. (2018). Psychological profile in coronary artery by-pass graft patients vs. valve replacement patients entering cardiac rehabilitation after surgery. Sci. Rep..

[21] Pakrad F., Ahmadi F., Grace S.L., Oshvandi K., Kazemnejad A. (2021). Traditional vs extended hybrid cardiac rehabilitation based on the continuous care model for patients who have undergone coronary artery bypass surgery in a middle-income country: A randomized controlled trial. Arch. Phys. Med. Rehabil..

[22] Pourafkari L., Ghaffari S., Tajlil A., Shahamfar J., Hedayati S., Nader N.D. (2016). The impact of cardiac rehabilitation program on anxiety and depression levels after coronary artery bypass graft surgery. Cor Vasa.

[23] Boban M., Persic V., Miletic B., Kovacicek K., Madzar Z. (2013). Heart surgery stems increased nutritional risk, expressed during the course of stationary rehabilitation. Ann. Nutr. Metab..

[24] Tramarin R., Pistuddi V., Maresca L., Pavesi M., Castelvecchio S., Menicanti L., De Vincentiis C., Ranucci M. (2017). Patterns and determinants of functional and absolute iron deficiency in patients undergoing cardiac rehabilitation following heart surgery. Eur. J. Prev. Cardiol..

[25] Wilkinson J.A., Harrison A.S., Doherty P. (2021). Obese patients’ characteristics and weight loss outcomes in cardiac rehabilitation: An observational study of registry data. Int. J. Cardiol..

[26] Conijn D., de Lind van Wijngaarden R.A.F., Vermeulen H.M., Vliet Vlieland T.P.M., Meesters J.J.L. (2022). Referral to and enrolment in cardiac rehabilitation after open-heart surgery in the Netherlands. Neth. Heart J..

[27] Pengelly J., Royse C., Williams G., Bryant A., Clarke-Errey S., Royse A., El-Ansary D. (2022). Effects of 12-week supervised early resistance training (secret) versus aerobic-based rehabilitation on cognitive recovery following cardiac surgery via median sternotomy: A pilot randomised controlled trial. Heart Lung Circ..

[28] Raghuram N., Parachuri V.R., Swarnagowri M.V., Babu S., Chaku R., Kulkarni R., Bhuyan B., Bhargav H., Nagendra H.R. (2014). Yoga based cardiac rehabilitation after coronary artery bypass surgery: One-year results on LVEF, lipid profile and psychological states—A randomized controlled study. Indian Heart J..

[29] Racca V., Rienzo M.D., Mazzini P., Ripamonti V., Gasti G., Spezzafferi Z. (2015). ICF-based approach to evaluating functionality in cardiac rehabilitation patients after heart surgery. Eur. J. Phys. Rehabil. Med..

[30] Pack Q.R., Dudycha K.J., Roschen K.P., Thomas R.J., Squires R.W. (2015). Safety of early enrollment into outpatient cardiac rehabilitation after open heart surgery. Am. J. Cardiol..

[31] Bianchi S., Maloberti A., Peretti A., Garatti L., Palazzini M., Occhi L., Bassi I., Sioli S., Biolcati M., Giani V. (2021). Determinants of functional improvement after cardiac rehabilitation in acute coronary syndrome. High Blood Press. Cardiovasc. Prev..

[32] Akpinar F.M., Oral A. (2023). Does exercise-based cardiac rehabilitation reduce mortality and hospitalization rates after heart valve surgery?: A Cochrane review summary with commentary. Am. J. Phys. Med. Rehabil..

[33] Yau D.K.W., Underwood M.J., Joynt G.M., Lee A. (2021). Effect of preparative rehabilitation on recovery after cardiac surgery: A systematic review. Ann. Phys. Rehabil. Med..

[34] Lin D.S.-H., Lo H.-Y., Yu A.-L., Lee J.-K., Chien K.-L. (2022). Mortality risk in patients with underweight or obesity with peripheral artery disease: A meta-analysis including 5,735,578 individuals. Int. J. Obes..

